# Particle Safety Assessment in Additive Manufacturing: From Exposure Risks to Advanced Toxicology Testing

**DOI:** 10.3389/ftox.2022.836447

**Published:** 2022-04-25

**Authors:** Andi Alijagic, Magnus Engwall, Eva Särndahl, Helen Karlsson, Alexander Hedbrant, Lena Andersson, Patrik Karlsson, Magnus Dalemo, Nikolai Scherbak, Kim Färnlund, Maria Larsson, Alexander Persson

**Affiliations:** ^1^ Man-Technology-Environment Research Center (MTM), Örebro University, Örebro, Sweden; ^2^ Inflammatory Response and Infection Susceptibility Centre (iRiSC), Faculty of Medicine and Health, Örebro University, Örebro, Sweden; ^3^ School of Medical Sciences, Faculty of Medicine and Health, Örebro University, Örebro, Sweden; ^4^ Department of Health, Medicine and Caring Sciences, Occupational and Environmental Medicine Center in Linköping, Linköping University, Linköping, Sweden; ^5^ Department of Occupational and Environmental Medicine, Örebro University, Örebro, Sweden; ^6^ Department of Mechanical Engineering, Örebro University, Örebro, Sweden; ^7^ Absolent AB, Lidköping, Sweden; ^8^ AMEXCI AB, Karlskoga, Sweden

**Keywords:** industrial 3D printing, particle emissions, adverse outcome, inflammation, genotoxicity, endocrine disruption, mechanism of action

## Abstract

Additive manufacturing (AM) or industrial three-dimensional (3D) printing drives a new spectrum of design and production possibilities; pushing the boundaries both in the application by production of sophisticated products as well as the development of next-generation materials. AM technologies apply a diversity of feedstocks, including plastic, metallic, and ceramic particle powders with distinct size, shape, and surface chemistry. In addition, powders are often reused, which may change the particles’ physicochemical properties and by that alter their toxic potential. The AM production technology commonly relies on a laser or electron beam to selectively melt or sinter particle powders. Large energy input on feedstock powders generates several byproducts, including varying amounts of virgin microparticles, nanoparticles, spatter, and volatile chemicals that are emitted in the working environment; throughout the production and processing phases. The micro and nanoscale size may enable particles to interact with and to cross biological barriers, which could, in turn, give rise to unexpected adverse outcomes, including inflammation, oxidative stress, activation of signaling pathways, genotoxicity, and carcinogenicity. Another important aspect of AM-associated risks is emission/leakage of mono- and oligomers due to polymer breakdown and high temperature transformation of chemicals from polymeric particles, both during production, use, and *in vivo*, including in target cells. These chemicals are potential inducers of direct toxicity, genotoxicity, and endocrine disruption. Nevertheless, understanding whether AM particle powders and their byproducts may exert adverse effects in humans is largely lacking and urges comprehensive safety assessment across the entire AM lifecycle—spanning from virgin and reused to airborne particles. Therefore, this review will detail: 1) brief overview of the AM feedstock powders, impact of reuse on particle physicochemical properties, main exposure pathways and protective measures in AM industry, 2) role of particle biological identity and key toxicological endpoints in the particle safety assessment, and 3) next-generation toxicology approaches in nanosafety for safety assessment in AM. Altogether, the proposed testing approach will enable a deeper understanding of existing and emerging particle and chemical safety challenges and provide a strategy for the development of cutting-edge methodologies for hazard identification and risk assessment in the AM industry.

## Introduction

Additive manufacturing (AM) is a manufacturing technology that in the last decade has completely revolutionized some industries and continue to do so in many sectors. AM, as opposed to subtractive manufacturing, such as machining, only utilizes the material that is needed for the component, with little waste material being produced. This is done by slicing a 3D model into thousands of layers and adding material in a layer-wise fashion to manufacture the component. Many different AM technologies exist, utilizing different processes and materials to build components. Some of the most common types of AM machines currently use the laser powder bed fusion (L-PBF) process, which use metal or polymer microparticulate powder in small fractions that is melted and fused locally by a laser (King et al., 2015). These machines offer close to unparalleled versatility and design freedom, making designs possible that were simply out of reach using conventional methods of the past. However, while this technology has come far in a short time, the industrial application of AM is a mere fraction of what the conventional technologies represent. Part of this delay is due to lack of experience and trust in the technology. To overcome this, the AM community of the Swedish industries have formulated a Strategic Research Agenda (SRA), in which key strategic areas in the field of AM have been concretized. On top of the list of the SRA are occupational health issues, design competence, and quality and productivity.

**FIGURE 1:**
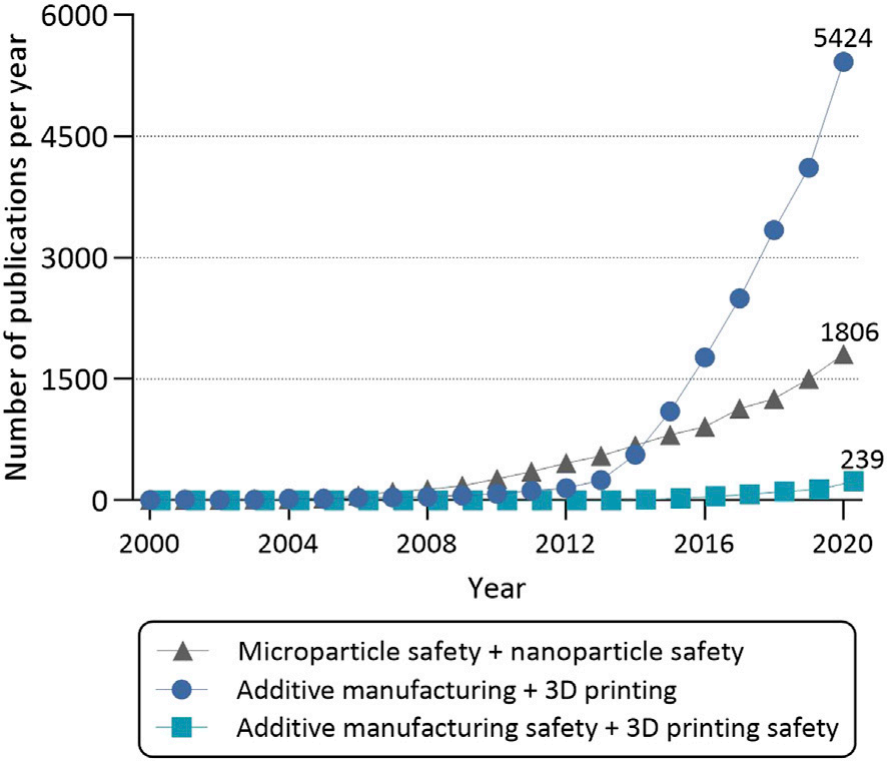
Overview of the papers published over the last 20 years on the topics “microparticle” and “nanoparticle safety,” “additive manufacturing” and “3D printing,” and “additive manufacturing safety” and “3D printing safety.” All publications were found *via* PubMed literature search.

“Those who cannot remember the past are condemned to repeat it”. Looking at the world today, the words by George Santayana are strikingly accurate. By all appearances, looking at the general lack of publicly available research concerning potential adverse health effects from working with AM, the words are true also for this field. As depicted in Figure 1, querying PubMed by using the search criteria “additive manufacturing” and “3D printing” reveals a striking increase of publishing on this topic over the last 20 years, reaching a peak in 2020 with 5,424 publications. At the same time, the number of publications investigating safety aspects of AM was growing rather slowly, reaching 239 publications in 2020. While the general understanding of the health effects imposed by exposure to different elements, such as nickel, cobalt, chromium, polymer additives, contaminants, and solvents, are known, knowledge is lacking on the byproducts and particle size ranges that are found in the AM industry. Long-term effects of low dose exposures are virtually unexplored territories. The same can be said about the exposure risks proposed by specific operations in the daily work, since measurements have been rarely done for particles at the nanoscale. By extension, no guidelines exist that describe the exposure limits or safety equipment requirements in AM, neither in Swedish nor in European Union’s regulation.

Particulate materials in AM technology are found in powder feedstocks for AM printers but can also be created during the AM printing process (Taylor et al., 2021). Therefore, inhalation and dermal exposure to feedstock particles or byproducts emitted from AM printers may occur at different stages of the production, from powder handling to printing post-processing, machine cleaning, and maintenance. In addition, metals applied mainly in directed energy deposition may be toxic or sensitizing. Furthermore, chemical fumes emitted during the operation of the AM printers could pose a significant risk for the occupational health and safety. Many volatile organic chemicals (VOCs) are known irritants with carcinogenic potential. Prolonged exposure to VOCs can cause eye, nose, and throat irritations, headache, or loss of coordination (Dobrzyńska et al., 2021).

The feedstock materials and particle emissions generated during the AM processes could vary greatly regarding toxicological potential, ranging from harmless to potential occupational health risks that could result in a future tragedy, as was seen with asbestos and silica. Therefore, this review will detail: 1) overview of the AM feedstock powders, impact of reuse on particle physicochemical properties, main exposure pathways and protective measures in AM industry, 2) role of particle biological identity and key toxicological endpoints in the particle safety assessment, and 3) next-generation toxicology approaches in nanosafety for safety assessment in AM. The herein proposed approach will enable a deeper understanding of existing particle and chemical safety challenges and provide a strategy for the development of cutting-edge methodologies for proactive hazard identification and risk assessment in the AM industry.

## Feedstock Powders in Additive Manufacturing

One of the most important production categories in the AM industry—L-PBF, such as selective laser sintering, electron beam melting (EBM), and selective laser melting (SLM)— utilize laser or electronic beam to selectively fuse polymeric, metallic, ceramic, or composite particle powders layer-by-layer into desired products according to their computer-aided design (CAD) models (Yuan et al., 2019). Table 1 provides a short overview of the most common polymeric, metallic, and ceramic powders applied in AM. In addition, it summarizes different types of reinforcements used in polymeric matrices.

**TABLE 1:** Chemical composition of powder particles applied in the AM industry-- > Class of particles/Chemical composition.

Class of particles	Chemical composition of particles	
Polymeric powders	Semi-crystalline thermoplastics	Polyamides (PAs), polypropylene (PP), polyaryletherketone (PAEK), polyethylene (PE), polybutylene terephthalate (PBT)
	Amorphous thermoplastics	Polycarbonate (PC) and polystyrene (PS), Acrylonitrile Butadiene Styrene (ABS)
	Thermoplastic elastomers	Ester-based polyurethane (PU)
	Biocompatible polymers	Polyvinyl alcohol (PVA), polycaprolactone (PCL), polyhydroxybutyrate-co-hydroxyvalerate (PHBV), polylactide (PLA), polyglycolide (PGA)
Metallic powders	Titanium alloys	Ti6Al4V (α-β titanium alloy)
	Nickel alloys	NiCr19Fe19Nb5Mo3 (precipitation nickel-base superalloy), NiCr22Fe18Mo
	Aluminum alloys	AlSi10 Mg (hypoeutectic Al–Si casting alloy), AlCu4Mg1, AlCu3.5LiAgMg, AlCu6Mn, AlMg4.5Mn0.7, AlZn6MgCu, AlZn4.5Mg1, Al5.6Zn-2.5Mg-1.6Cu-0.23Cr
	Steel alloys	22Mo9Nb, X5CrNiCuNb 16–4, X2CrNiMo 17–12-2, 25CrMo4, 16MnCr5, 42CrMo4, X5CrNiCuNb 17–4, X3NiCoMoTi 18-9-5, X40CrMoV5-1
	Cobalt alloys	CO212, CO502, CO90, Co49Fe2V
	Copper alloys	OFHC Cu, HC Cu, Cu10Al, Cu10Sn, Cu15Sn
Ceramic powders	Oxide and non-oxide advanced ceramics	Alumina (Al_2_O_3_), zirconia (ZrO_2_), silicon carbide (SiC), tungsten carbide (WC), boron carbide (B_4_C), silicon nitride (Si_3_N_4_), aluminum nitride (AlN), zirconium diboride (ZrB_2_)
	Polymer-derived ceramics	SiC, Si_3_N_4_, silicon oxynitride (SiON), silicon oxycarbide (SiOC), silicon carbonitride (SiCN), boron nitride (BN) and boron carbonitride (BCN)
	Ceramic matrix composites	Carbon fibers/carbon matrix (C_f_/C), C_f_/SiC matrix, SiC_f_/SiC
Reinforcement of polymeric matrices	Metallic fillers	Aluminum and carbon steel
	Ceramic/glass fillers	Silica, glass beads, clays, and oxides
	Carbon-based fillers	Carbon black, carbon nanotubes (CNTs), graphite and graphene
	Organic additives	Polycarbonate (PC) and polystyrene (PS)

## Impact of Reuse on Particles’ Physicochemical Properties

Importantly, changes in the particle physicochemical features, including size, shape, porosity, surface topography, interfacial free energy, as well as chemical composition and surface oxidation can profoundly affect biochemical mechanisms when particles arrive at the biological interface (Rahmati et al., 2020). This aspect of particle safety assessment is crucial in classifying materials based on their toxic potential.

The reuse of powder in AM may affect the physiochemical properties of the powder particles. In literature, the terms reuse and recycling have been used interchangeably. However, recycling refers to the production of new atomization feedstock by remelting scrap materials or recover metal from other manufacturing methods. Reuse of feedstock powder is done when a single powder batch is used repetitively in an AM machine through multiple cycles until the powder is out of specification or predefined boundaries. In this section of the review, reused powder particles of commonly used metals in AM, such as stainless steel, alloys of aluminum, titanium and nickel-based alloys are in focus.

There are several factors, such as material type and number of reuse cycles, that may affect the physiochemical properties of the powder particles. Slotwinski et al. (2014) noted the presence of metal oxides on both virgin and reused 17-4 SS stainless steel powder particles. The powder was reused eight times in a L-PBF process. However, the study showed that there was no significant difference between the virgin and reused powder particles in terms of metal oxide type and concentrations. Gorji et al. (2019) investigated the reusability of 316L stainless steel powder, reused over 10 times in the L-PBF process. The study showed that the reused powder had greater surface oxidation and higher concentration of metallic oxides. Heiden et al. (2019) discovered significant changes in surface composition, oxide thickness, and magnetic properties of reused 316L stainless steel powder particles. Virgin and reused powder were investigated, and the powder was used through 30 build cycles in the L-PBF process. They showed that surface oxygen content, iron oxide Fe_3_O_4_ thickness, powder magnetic susceptibility and magnetic moment increased with reuse, the latter due to increase in δ ferrite. Similar findings were reported in a more recent study by Wang et al. (2021), where powder particles, reused at least once, were investigated.

Another metal, commonly used in AM, that has high tendency for surface oxidation is aluminum. An oxide layer of Al_2_O_3_ is formed when oxygen is chemisorbed and, therefore, correct Al-based powder handling is crucial to avoid oxidation (Nie et al., 2016; Riener et al., 2021). Cordova et al. (2019) displayed an increase of oxygen content from 0.005 to 0.01 wt% after six reuse cycles of gas atomized AlSi10Mg powder in a L-PBF process. Inconel 718 and Ti6Al4V were also included in the study, but the oxygen content only varied for the reused AlSi10Mg powder. In contrast, Maamoun et al. (2018) showed that the chemical composition and surface oxide content were identical for virgin and reused AlSi10Mg powder after 18 reuse cycles in L-PBF. They proposed that AlSi10Mg powder could be reused if proper sieving was applied.

**FIGURE 2:**
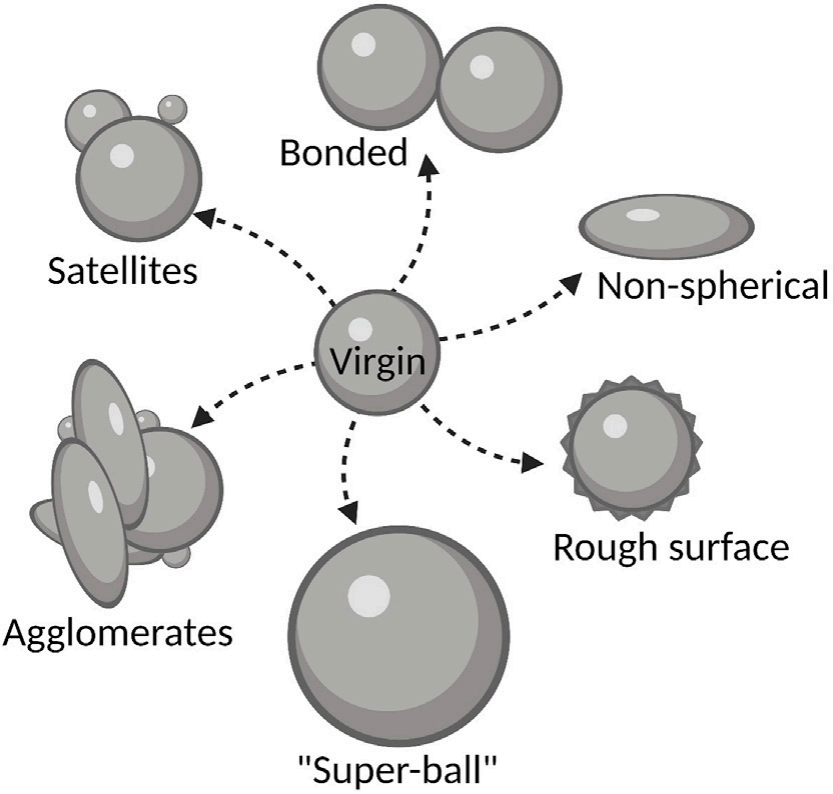
Impact of reuse on the properties of microparticle feedstock powders in AM. Large energy input during production usually elicits formation of satellites (small particles attached to the surface of bigger particles), bonded particles, non-spherical and elongated particles, tightly bound agglomerates, particles with rough/irregular surface topography or “super-ball” particles with increased size in comparison to the virgin particles.

Titanium is a highly reactive metal toward oxygen, and Nandwana et al. (2016) showed that the oxygen content increased when Ti6Al4V metal powder was reused five times in an EBM process. Similar findings were reported by Tang et al. (2015). They studied reused Ti6Al4V metal powder through 21 build cycles and showed that the oxygen content increased progressively with powder reuse cycles. In addition, Popov et al. (2018) showed that after many reuse cycles, Ti6Al4V metal powder particles display a variety of defects, including satellites, bonded particles, elongated and non-spherical particles, “super-balls,” and particle agglomerates (Figure 2).

In addition to Ti6Al4V, Nandwana et al. (2016) included the nickel-based alloy Inconel 718 in the study on powder reusability in EBM. In contrast to Ti6Al4V, only minor changes in chemistry of the virgin and reused powder were found, and the oxygen content increased from 0.014 to 0.016 wt%. Similar findings were reported by Gruber et al. (2019) as the average oxygen level increased from 146 ppm to 266 ppm after 14 build cycles. Additionally, they observed Al-rich nanosized particles forming on the surface of the powder already after the first reuse cycle. The particles tended to grow with increasing number of build cycles. Ardila et al. (2014) investigated the reuse of Inconel 718 in the L-PBF process but reported no significant change in chemical composition other than a minor oxidation of nickel. Wang et al. (2021) showed that there was no, or only minor, differences in surface oxide composition and metal release pattern of reused powder in a comparison to virgin powder in their study of reusability of Inconel 718 and 18Ni300, utilizing L-PBF.

## Additive Manufacturing Emerging Safety Challenges: Particle and Chemical Emissions

**FIGURE 3:**
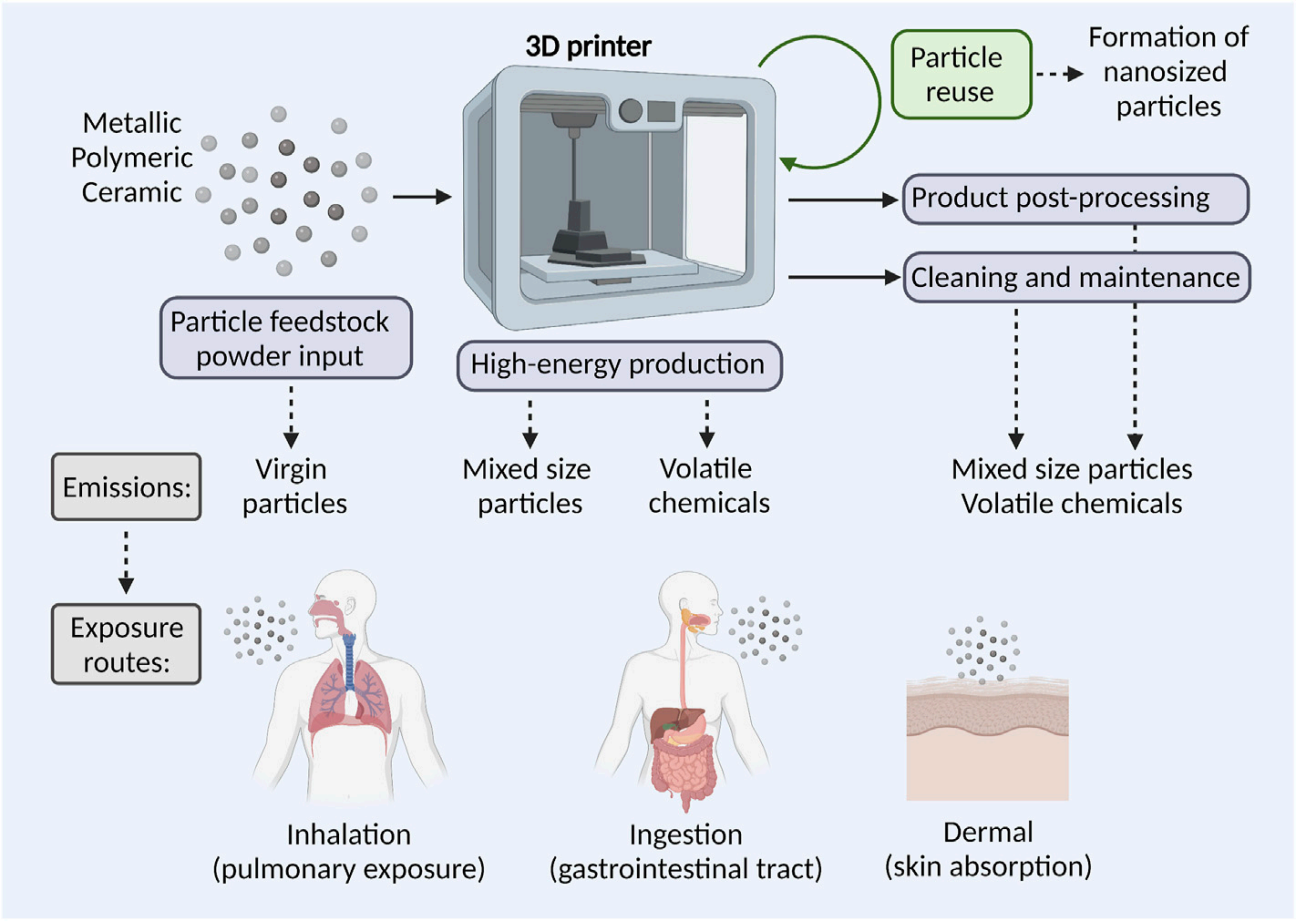
Particle and chemical exposure risks in AM. At different stages of the AM process chain particle and chemical emissions may occur, including input of feedstock powders, high-energy production, product post-processing, machine cleaning, and maintenance. Potential particle exposure routes for exposed AM workers involve inhalation, ingestion, and skin absorption.

As all work environments, AM work environments are controlled by occupational hygiene regulations to ensure the workers safety. Regarding airborne particles and chemicals, gravimetric or VOC analyzing techniques are today used to ensure that threshold limit values for specific compounds in air are not exceeded (Ljunggren et al., 2019; Runström-Eden et al., 2021). However, it has been shown that the gravimetric measurements are not sufficient when assessing exposure-related health risks; mainly because particle sizes vary from 10 nm to 65 µm or larger, and due to different AM work activities entail different emissions and thereby exposure risks. Therefore, particle-counting instruments are suggested as complement to gravimetric measurements, mainly to be able to identify particularly hazardous process steps (Ljunggren et al., 2019). Exposure assessment, health hazards of particles, possible chemical hazards as well as implications for risk assessment and management in metal AM work environments have recently been reviewed (Chen et al., 2020; Dobrzynska et al., 2021; Leso et al., 2021). An increasing body of evidence supports that the micro and nanosized particles and/or VOCs are present in the AM environments. Figure 3 depicts possible particle emission sources in the AM process chain and the most common human exposure pathways.

### Pathways of the Particle Emissions

Open metal powder handling, such as powder input into a machine that is not equipped with an enclosed powder handling system, entails particle emissions in sizes from 10 nm up to the largest declared sizes of the feedstock powder. In addition, an increase of micro and nanosized particles has been found in reused Inconel 939 powder after SLM printing, compared to virgin powder (Mellin et al., 2016; Graff et al., 2017). Studies of high-energy techniques, such as SLM, have indicated that the nanosized particles are created during the AM process; with a subsequent release into the working environment when the chamber door is opened (Ljunggren et al., 2019). Information is still limited regarding exact number and sizes of particles emitted at specific AM process steps but when studying SLM printing with Hastelloy X alloys, Ljunggren et al. (2019) found that most gravimetric analyses performed in the metal AM facilities were within occupational exposure limits (OELs). The background number of particles, with an average size of 50–100 nm, were <20,000 particles/cm^3^, which is rather low compared to other metalworking facilities or outdoor environments. However, elevated numbers of nanosized particles (50–100 nm) were detected during post processes, such as sawing of printed products from the construction plate or de-powdering of finished products, reaching approximately 50,000 particles/cm^3^. Even though the levels of micro and nanosized particles are substantially lower in AM than for example welding environments, the importance not to neglect the larger inhalable particles (<15 µm) present in AM environments must be pointed out - as they as well may pose a health risk. Importantly, a study of health effects in the same AM workers, as described above by Ljunggren et al. (2021), confirmed biological uptake of metals present in the powders.

Regarding particle emissions in polymer printing environments, a study by Väisänen et al. (2019) showed that particle concentrations were highest (2,070-81,890 particles/cm^3^) during manufacturing with methods where plastics were thermally processed. Another study by Zisook et al. (2020) showed that submicron particles, predominantly nanoparticles, were produced during material extrusion printing using ABS at approximately 12,000 particles/cm^3^ above background. After subtracting the mean background concentration, the mean concentration for material extrusion printing operations correlated with a calculated emission rate of 2.8 × 10^10^ particles/min^−1^ under the conditions tested. During processing of parts produced using material jetting (MJ) or L-PBF, particle emissions were generally negligible. This indicates that airborne emissions associated with AM operations are variable, depending on printing and parts handling processes, raw materials, and ventilation characteristics. In addition, Runström-Eden et al. (2021) have recently studied particle and VOC emissions from four different printing techniques: L-PBF, material extrusion (ME), MJ, and vat photopolymerization. The most significant emissions of particles in the size range 10 nm to 1 µm were found during ME printing. Background levels before the start of the printer were between 5,000 and 10,000 particles/cm^3^, while particle numbers outside the printer during printing ranged between 5,000 and 15,000 particles/cm^3^ with isolated peaks at 50,000 particles/cm^3^. Inside the printer hood, emissions of 500,000 particles/cm^3^ were detected, and during post processing, emissions of 75,000-300,000 particles/cm^3^ were found; indicating that negative exposure-related health effects cannot be ruled out.

### Pathways of the Volatile Chemical Emissions

VOCs are organic substances with a boiling point of 50–320°C. Aromatic and aliphatic hydrocarbons, esters, ketones, and aldehydes constitute this group of substances. These compounds are used in solvents, paints, glues, degreasing agents, etc. but are also part of gasoline and other mineral oil products. In the AM industry, the materials entering the machines may release VOC gases or vapors, especially when using polymeric materials heated during the printing process. The printed products might be warm when taken out of the printers; thereby continue to emit VOCs. The types of VOCs that would be emitted depends on the printing material, printing method, and the pre- and post-activities. When, for example, using ABS plastic material in AM, the main substance emitted from the printers was styrene (Dobrzyńska et al., 2021). Other VOCs identified during AM when using ABS plastic material were acrylonitrile, acetonitrile, methyl methacrylate, propylene glycol, methyl styrene, cumene, cyclohexanone, ethylbenzene, toluene, butanol, and acetone (Wojtyla et al., 2020). In addition, post-processing of the 3D-printed products, including excess material removal, curing, heat-treatment, support removal, machining, surface finish processes (e.g., bead-blasting), vapor smoothing (using acetone, ethyl acetate, etc.), coloring, can also lead to the potential VOC exposure. Exposures to VOCs is of concern for workers because some of these chemicals are respiratory and mucous membrane irritants (He et al., 2015).

Opening industrial-scale FDM™ 3D printer doors after printing, removing desktop FDM™ 3D printer covers during printing, acetone vapor polishing (AVP) and chloroform vapor polishing (CVP) tasks all resulted in transient increases in levels of VOCs. Personal exposures were 380 to 6,470 μg/m^3^ for acetone during AVP and 180 μg/m^3^ for chloroform during CVP (Du Preez et al., 2018). Väisänen et al. (2019) showed that VOC concentrations, were low (113–317 μg/m^3^) during manufacturing where plastics were thermally processed, and during vat photopolymerization. However, Zisook et al. (2020) showed that total VOC concentrations of MJ and multi jet fusion methods were higher (1,114–2,496 μg/m^3^), probably due to material and binder spraying, where part of the spray can become aerosolized. Chemical treatment of 3D-printed objects was found to be a severe VOC source as well. Formaldehyde was detected in low concentrations (3–40 μg/m^3^) in all printing methods except for MJ, in addition to several other carbonyl compounds. Measurements by using VOC sensors at four Swedish AM facilities using polymeric printing materials and different printing methods showed the varied levels of VOCs during different production activities. When comparing the different printing techniques, and feedstock materials, MJ had the highest concentration of VOC (3,200 µg/m^−3^). High peak exposures, 18,000–99,000 μg/m^3^, were measured during cleaning of the printer when post-processing printed material (Runström-Eden et al., 2021). Importantly, an increase in VOC was observed in the evening and a decrease in the morning, roughly the same time as the changes in ventilation settings occur. This finding strengthens the fact that AM facilities should implement adequate preventive measures (high-efficiency filters in AM printing machines, encapsulated processes, printers with hoods, etc.) to reduce particle and VOC exposure risks.

## Protective Measures in Additive Manufacturing: Case of Industrial Filters

The particles used in AM are often in the range of 20–100 μm, but the emissions are primarily from used and degraded particles of smaller size. Therefore, it is necessary to use high efficiency particle filters in the printing machines, at least of class H13, as final filters to guarantee a separation efficiency of 99.95%. However, it is necessary to restrict the air speed at low levels, since efficiency is strongly related to air speed when the major capture mechanism for nanosized particles in fiber filters is diffusion due to Brownian motion (Nelson, 2020). It is also important to have an indoor filtration system with high air output that prevent accumulation of small particles and gases in the indoor air.

**FIGURE 4:**
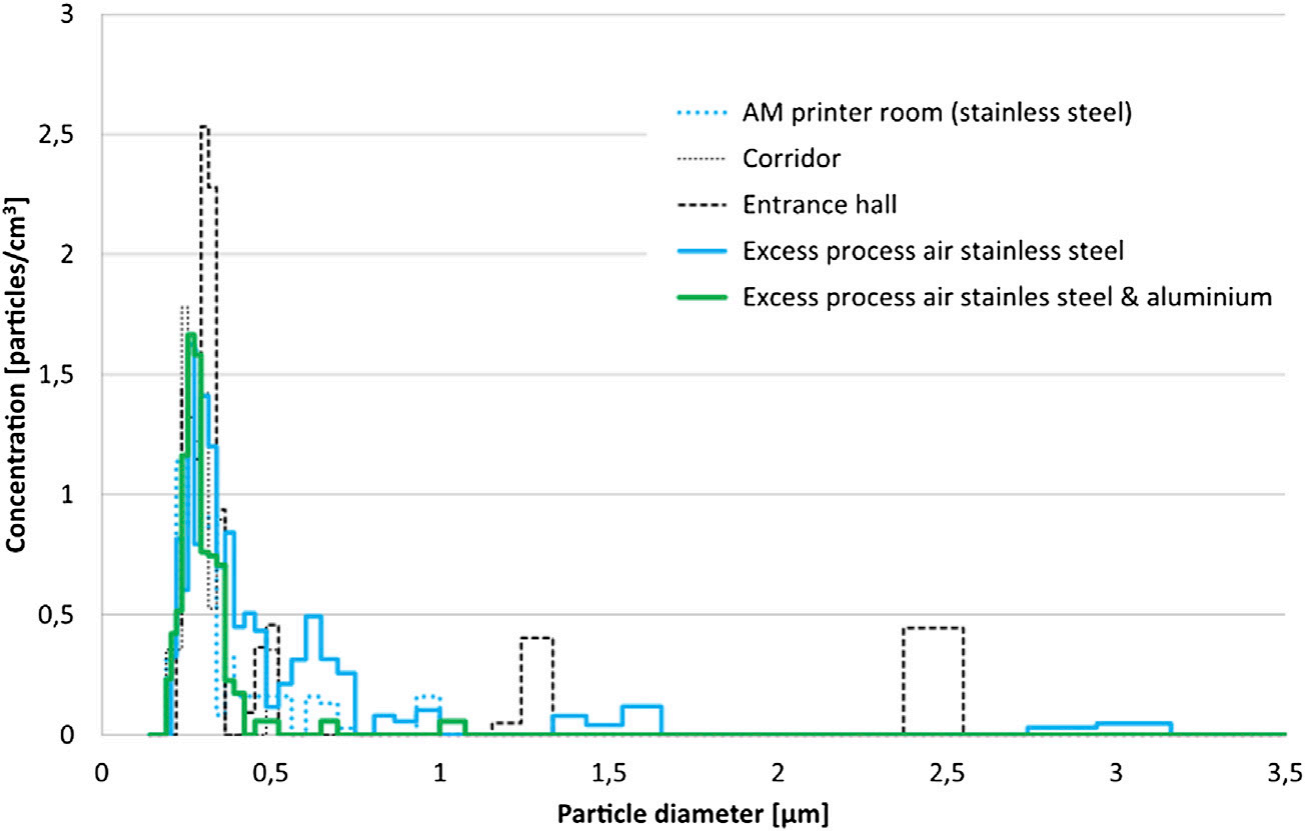
Particles in the AM occupational setting. The particle size distribution was quite similar in all measurement locations. Some larger particles (>1 µm) could be found in the entrance hall and in the process air.

For example, AM printing machines that process stainless steel and aluminum have dust cartridges that are cleaned by compressed air pulses, to keep the concentration of particles low in the process air. It is primarily argon gas that is circulating from the machine to the filters and *vice versa*. Excess process air is first passing through a hepa H13 filter before it is released outside the building. It is difficult to perform any internal measurements of the process since the system has a pressure of 0.5 bar and sensors will shut off the machine if the pressure decreases. However, our preliminary measurements of external printer air from one AM facility (unpublished data) show that the particle concentration (Ø >0.15 µm), measured with Welas 2000 (Palas instrument), was low in all measuring locations and the same was observed for the larger particles above 1 µm (Figure 4). Parallel measurements with the NanoTracer (Philips) indicated higher concentration of nanosized particles, with an average particle diameter of 80 nm. This supports that nanosized particles are emitted in the AM occupational setting, and it is therefore of utmost importance to setup working layouts, including air-ventilation with proper filters, to ensure protection and reduce exposure risks for the AM workers.

## Impact of Particles’ Properties and Acquired Biological Identity on Cell Recognition and Uptake

Understanding the interaction of particles with a biological system (i.e., biomolecules, organelles, cells, tissues) is a fundamental challenge in the particle safety research (Boraschi et al., 2020; Himly et al., 2020). The outcome of these interactions governs particle fate, including recognition, internalization, distribution within the cells, and activation of different signaling pathways.

**FIGURE 5:**
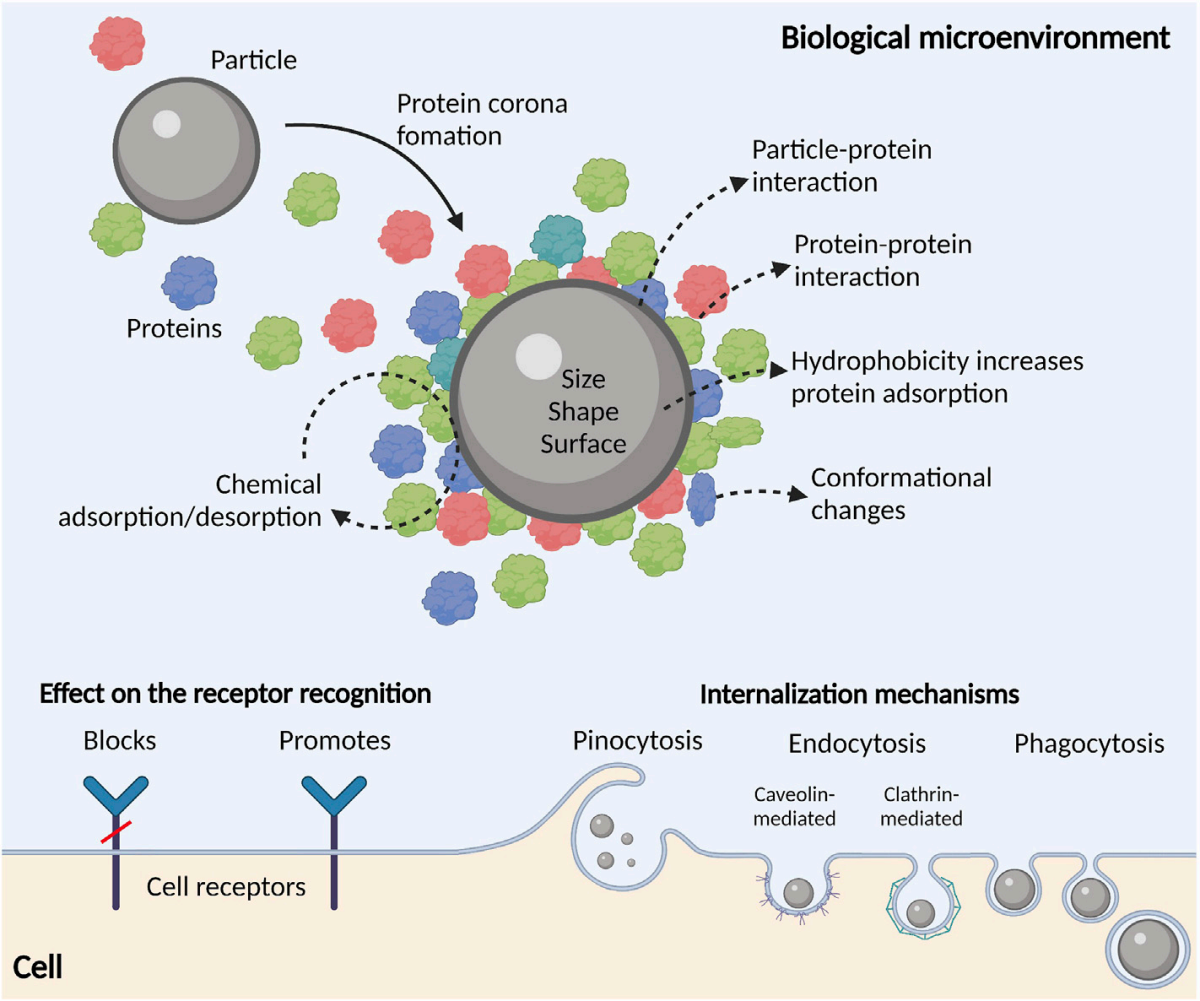
Particle’s biological identity. Particles entering protein-rich biological environments are swiftly covered by proteins forming the so-called protein corona or mechanical interface between particles and cell receptors. Particle physicochemical properties are directly dictating the composition of the protein corona and the extent to which proteins adsorb/desorb from the particle’s surface. The protein corona may block or promote receptor recognition and particle internalization by the cells. Particle’s size determines the mechanism of the internalization, and it may involve pinocytosis, endocytosis (caveolin- or clathrin-mediated) and phagocytosis.

When the particle enters the biological microenvironment (i.e., blood, lung fluid or experimental cell culture media), its surface is covered by a layer of adsorbed proteins that form a so-called protein corona (Figure 5). The protein corona alters the particle size, aggregation potential, and surface chemistry, giving the particle a biological identity that is different from its synthetic physicochemical identity (Ke et al., 2017). The corona further determines the biological responses by mediating the interaction of the particle with biomolecules (proteins, metabolites), membrane receptors, and physical barriers (lung epithelial cells, endothelial cells). The protein corona is a direct function of the particles’ physicochemical properties, biological microenvironment, and the duration of exposure (Lundqvist et al., 2008). Each biological microenvironment has a distinct set of proteins that interact in a specific way with the particles’ surface. Protein adsorption to particles does not always require direct contact with the particle surface and may instead occur *via* protein-protein interactions (Li and Lee, 2020). In addition, proteins can undergo conformational changes that together with nonspecific protein-protein interactions may be interpreted as a danger-associated signal (Corbo et al., 2016); initiating an immune response that may develop into a successful defensive response or an uncalibrated inflammatory reaction (Boraschi et al., 2020). Moreover, proteins adsorbed to a particle surface are in a constant state of flux, thereby causing changes in the composition of the protein corona because of continuous desorption/adsorption or by the so-called Vroman effect. The Vroman effect states that the adsorbed proteins can be replaced during time by proteins with higher affinity for the particle surface, even when present in smaller amount (Angioletti-Uberti et al., 2018).

As protein adsorption occurs at the interface between the particle and the biological microenvironment, surface charge, topography, surface area, and size, are features governing protein interactions (Richtering et al., 2020). Notably, hydrophobic particles with charged surfaces tend to adsorb more proteins, causing their denaturation to a greater extent (Pustulka et al., 2020). For example, polystyrene nanoparticles with increasing negative charge and hydrophobicity increase the total protein adsorption (Rahman et al., 2013). Research has also found that protein binding capacity of the particle surface positively correlates with the rate of particle internalization by cells. For example, particles that easily adsorb plasma proteins strongly interact with tissue-resident macrophages, causing rapid blood clearance and accumulation in the liver and spleen. A special subset of plasma proteins called opsonins (Wright and Douglas, 1904; Cockram et al., 2021), facilitate the recognition and internalization of different particles by macrophages (Mirshafiee et al., 2016). Still, protein adsorption can have the opposite effect on the internalization as the adsorbed proteins in some cases can inhibit the interaction between cellular receptors and the encountered particle surface (Cai et al., 2019).

Generally, particles are readily internalized by phagocytic cells. In some cases, it has been shown that pattern recognition receptors (PRRs), such as scavenger receptors (SRs) and Toll-like receptors (TLRs) participate in the recognition and internalization, and NOD-like receptors (NLRPs) interact with particles following internalization, and it is crucial to note that the properties of the protein/biomolecular corona dictate the type of interaction between particles and PRRs (Boraschi et al., 2020). Another important aspect influencing the particle fate is size. Increase in the overall size of the particle, alters protein adsorption capacity and subsequent interaction potential with biological barriers (Clift et al., 2008; Walkey et al., 2012). For example, particles with a diameter ≤500 nm as well as rod/fiber-shaped particles were preferentially taken up by dendritic cells compared to larger particles or those with cubic or spherical shape (Casals et al., 2011). Herd et al. (2013) investigated cellular uptake of three silica particle constructs: worm-like (232 × 1,348 nm), cylindrical (214 × 428 nm), and spherical (178 nm). They found that the internalization rate depended on particle geometry. The authors linked the variations they found to the different internalization mechanisms undergone by particles with different geometries. Smaller particles (≤500 nm) are mainly internalized *via* pino or micro-pinocytosis and *via* clathrin- or caveolin-mediated endocytosis, while particles larger than 500 nm are mainly internalized by phagocytosis (Behzadi et al., 2017; Foroozandeh and Aziz, 2018). In this context, high-energy input and powder reuse in AM significantly alter particle physicochemical properties and by that, most probably, change particle fate, recognition, and cellular responses.

By focusing on particles’ physicochemical characteristics and growing body of safety studies, materials scientists now have a better grasp on the relationships between the particles’ physicochemical features and their hazard/safety profiles. Hence, it is expected that an integration of design synthesis and safety assessment will foster particles safer-by-design by considering both applications and later safety/hazard implications (Morose, 2010; Lin et al., 2018). Proposed strategies to reduce hazard for particles comprise coating, control of size, doping, managing shape and crystallinity, reducing the presence of substances at the surface of particles that contribute to hazard, reduced persistence, and substitution (Geraci et al., 2015; Reijnders, 2020). Until today, a few safer-by-design strategies that have been implemented to make safer metal oxide, carbon-based, silica, and rare Earth oxide nanoparticles. These examples demonstrated the key aspect of particles safer-by-design, i.e., knowing which physicochemical characteristics contributed to toxicity and how to remediate them by rational design (Lin et al., 2018). This approach may have significant implications when designing novel materials for the AM.

## Toxicological Endpoints in the Particle Safety Assessment

### Inflammation

The correlation between occupational and environmental particle exposure and human respiratory disease has been known for a long time and in 1995 Seaton et al. (1995) put forward a hypothesis that lung exposure to particles mediated alveolar inflammation as a central common denominator for systemic effects, including cardiovascular disease (CVD). This idea was further developed by Sjögren (1997) describing the connection between occupational dust exposure, inflammation, and ischemic heart disease. In addition to effects on hormonal and blood pressure regulation, particle exposure is known to generate both local and systemic inflammatory responses, where the systemic inflammatory reactions are likely contributing to the strong relationship between particle exposure and CVD (Golia et al., 2014). While extensive and acute exposure can lead to bursts of proinflammatory mediators, it is the low-grade inflammation due to long-term exposure that is most associated with particle exposure. It is further important to note that nanosized particles, in comparison to larger inhalable fractions, may leak into the blood circulation and cause direct effects in tissues—distant from the immediate exposure site, including organs and blood vessel walls (Miller et al., 2017).

Focus of this review section is on the respiratory inflammation. In that context, particle size is an important factor for their toxic potential that affects two critical factors: the main region of exposure in the respiratory tract, and the surface area of the particles. As already mentioned, the surface of a particle plays a detrimental role in its toxicity, allowing interactions of the particle to biological structures, such as membranes and proteins. Therefore, higher surface area to mass ratio potentiates the toxicity of a particle. The size also determines how far the particles can reach in the respiratory tract, and the capacity to leave the deposition tissue and exit into the blood stream. The respiratory tract can be divided into three regions for particle exposures: 1) the upper respiratory tract, also called the extrathoracic region, comprising the mouth and nasopharynx, 2) the tracheobronchial or thoracic region, comprising the trachea, main bronchi, and bronchi of the conducting airways, and 3) the alveolar region, comprising the bronchioles and alveolar sacs and ducts. The particle fractions corresponding to each respiratory region include: 1) the inhalable fraction ( <100 µm) representing particles mainly reaching nose and mouth, and 2) the thoracic fraction ( <10 µm) that can pass the larynx, and the respirable fraction ( <4 µm) that can reach the alveoli. For studies on ambient particle exposures, PM2.5 is often used, which corresponds to the respirable particle fraction. In addition, the ultrafine particle fraction corresponds to the nanoparticle range of particles less than 100 nm in size. For further reading, the current sampling criteria to assess exposures of the different respiratory regions is thoroughly reviewed by Vincent (2005).

Deposition of the ultrafine or nanosized particles in the respiratory tract is not uniform but is size dependent. The total deposition increases as the size decreases, reaching near 100% deposition for 1 nm particles. However, for the smallest particles, the main deposition is in the extrathoracic region, in contrast to particles in the 10–100 nm range for which deposition in the alveolar space dominates (Geiser and Kreyling, 2010). It is also important to consider that the actual particle size can be affected by agglomeration of nanoparticles and adsorption of water molecules (Szałaj et al., 2019). Nanosized particles may further cross cellular membranes, leave the alveolar space through the alveolar epithelium and reach the bloodstream, where they may cause adverse effects on the cardiovascular system as well as distant organs (Geiser et al., 2005; Schulz et al., 2005; Miller et al., 2017).

#### Mediators of Inflammation

Inflammation is closely associated with the soluble factors, chemokines, cytokines, alarmins, lipid mediators, reactive oxygen/nitrogen species and acute phase proteins modulating the response, and these factors provide useful tools for the evaluation of the immunogenicity/immunotoxicity of particles. Cytokines and chemokines have central modulating effects to the activity of many cells and can be produced by most cell types in our bodies but are most strongly correlated with immunocompetent cells (Turner et al., 2014). Inflammatory mediators are pleiotropic in nature, and depending on the signaling landscape in the microenvironment, many effects overlap, synergize, and antagonize each other. In general, high levels of the proinflammatory mediators are associated with immunological activation to the degree that it is immunotoxic and cell and tissue damaging. Soluble inflammation markers are readily quantifiable with standard techniques, including antibody-based immunoassays, whereas lipid mediators, with some exceptions, require a mass spectrometric approach for detection.

Inflammasomes are multiprotein complexes that are composed upon cellular sensing of danger. Among the various inflammasomes, NLRP3 inflammasomes are the best described and their activity results in the activation and release of Interleukin-1β (IL-1β) and Interleukin-18 (IL-18), among the most potent proinflammatory soluble factors identified. The NLRP3 inflammasome can be activated in response to various stimuli, including extracellular ATP, K^+^ ionophores, heme, pathogen-associated RNA, bacterial and fungal toxins and/or components, as well as endogenous and exogenous particulate matter (Kanneganti et al., 2006; Mariathasan et al., 2006; Martinon et al., 2006; Hornung et al., 2008; Lee et al., 2016). The NLRP3 inflammasome has been found to be activated by organic, inorganic, metal, and elemental nanosized particles of various shapes (Schanen et al., 2009; Meunier et al., 2012; Yang et al., 2012). The dispersion state and physicochemical characteristics of the particles, such as size, aspect ratio, and surface charge and functionalization, are important factors to determine how potent the particle is in provoking NLPR3 inflammasome activation (Sun et al., 2013; Wang et al., 2017). Mechanistic studies revealed that NLRP3 inflammasome activation induced by long aspect ratio nanomaterials involves lysosomal damage, and subsequent cathepsin B release that serves as a signal for the assembly of the NLRP3 inflammasome (Wang et al., 2012). The size effect of particles on NLRP3 inflammasome activation has been examined by several studies, though contradictory results were obtained. Sharp et al. (2009) compared polystyrene particles of 430 nm, 1 µm, 10 µm, and 32 µm in diameter in inducing IL-1β release in dendritic cells and showed that smaller particles were more potent in promoting IL-1β production due to efficient internalization (Sharp et al., 2009). Inflammasome activation can be determined through an array of assays; ranging from study of composition of the complex microscopically or detection using western blot, and quantification of the active protease activity (caspase-1) using fluorescent probes coupled to a caspase-1 cleave site.

#### Cell Death and Immunomodulation

Many of the cellular responses to particle exposures are dependent on uptake and internalization of the particle, e.g., through phagocytosis, endocytosis, or pinocytosis. The cellular response following uptake is believed to be governed by the size and shape of the solid particle, where sharp and spiky shapes can cause damage to membranes and thus result in a greater impact on the cell responses compared to spherical and rod-shaped particles. Also plate shaped particles (nanosheets) has been shown to exert a more potent toxicity than more conformed shaped particles (Cai et al., 2018; Xu et al., 2020). Much of the described adverse cellular responses are related to interaction with, or destabilization of, cellular membranes either by oxidation or other interaction with phospholipids or sheer abrasive stress on the membrane. Following internalization of particles, lysosomal destabilization with cathepsin leakage into the cytosol has been described as a major effect in particle-induced cellular events, involving mitochondrial membrane destabilization and ROS generation, often leading to cell death. In addition, in neutrophils, a wide range of particles have been described to provoke formation of neutrophil extracellular traps (NETs) (Desai et al., 2017). NETs were initially described as an effective weapon against bacteria, fungi, viruses, and further pathogens, a list now supplemented with recent discoveries that particulate matter is capable of inducing NETs formation (Brinkmann et al., 2004; Skallberg et al., 2019).

#### Clinical Samples

To investigate the direct effects of exposure to the human body, investigations of inflammatory markers in exposed people, e.g., in occupational settings is often the approach. However, low exposures are generally not reflected in readouts of acute inflammation, such as chemokines and proinflammatory cytokines, C-reactive protein (CRP), or in cellular readouts, such as inflammasome activity and ROS. Dilution effects makes it also challenging to measure local mediator production in e.g., serum, where the factors present in the circulation are of tissue origin and have thus been extensively diluted. The local effects in the exposed tissue microenvironment may however still contain biologically relevant levels of mediators but this is most often out of reach for today’s investigations.

Direct lung measures, including fractional exhaled nitric oxide (FeNO) is an established measure of inflammatory activity in the airways. NO can be produced by most immunocompetent as well as tissue cells in the lung as a response to endogenous mediators, such as chemokines and cytokines as well as by exogenous irritants, e.g., as bacterial toxins, viruses, or allergens (Watkins et al., 1997; Pechkovsky et al., 2002). In a study by Gümperlein et al. (2018), 26 healthy adults in a single-blinded, randomized, cross-over design were exposed to emissions of a desktop 3D printer using fused deposition modeling (FDM) for 1 h (high UFP-emitting ABS vs. low-emitting polylactic acid (PLA)). The results showed difference in the time course of FeNO, with higher levels after ABS exposure.

The non-ciliated Club cells (formerly known as Clara cell), containing the most P-450 activity in the lungs, are known for their vulnerability to toxic insults. These cells are the source of the lung epithelium specific biomarker Club cell secretory protein 16 (CC16), also known as CC10 and uteroglobin. CC16 exerts anti-inflammatory properties involved in the airway inflammatory homeostasis, and the molecule can passively diffuse across the bronchoalveolar-blood barrier into serum (Milne et al., 2020). Elevated concentrations of CC16 in serum and urine thus provide a useful biomarker for evaluation of the integrity of the lung epithelial barrier and airway inflammation (Arsalane et al., 2000), whereas diminished levels indicate severe lung injury (Kropski et al., 2009). The correlation between PM2.5 exposure and elevated CC16 levels in serum and urine of have been indicated in several studies; however, some studies report inverse correlations possibly due to the homeostatic nature of the inflammatory factor as well as differences in the content and composition of the particulate matter (Timonen et al., 2004; Jacquemin et al., 2009; Vattanasit et al., 2014; Andersson et al., 2019).

Further, standard clinical measurements, such as total white blood cell counts and 5-part differential counts (neutrophils, lymphocytes, monocytes, eosinophils, basophils) providing neutrophil-to-lymphocyte ratio (NLR) as well as the lymphocyte-to-monocyte ratio (LMR) may further provide useful tools bearing evidence of the result of elevated systemic inflammatory status able to predict cardiovascular disease events (Prats-Puig et al., 2015; Huang et al., 2018).

#### Ex vivo

When quantification of factors in serum, urine, or exhaled air is not sufficient to evaluate the inflammatory status, *ex vivo* experimentation of cells isolated from exposed individuals may provide valuable insight into the condition of the cellular components. One approach is to extract cells from exposed individuals, e.g., through blood sampling and cell isolation, to investigate the cellular responses in an isolated model. By this, the cells inflammatory activity can be evaluated directly, for instance by flow cytometric quantification of the monocyte activation marker CD11b, which has been found to positively associate with PM2.5 exposure (Karottki et al., 2015). The *ex vivo* approach offers a possibility to study not only soluble mediators, but also detection of changes of the cellular inflammation, including inflammasome activation, CD-marker, and receptor expression as well as inflammation-associated readouts, such as mitochondrial depolarization, ROS generation, lysosomal destabilization, acidification, and cell death in a certain cell population. In addition, the extracted cells can further be experimentally provoked by additional inflammatory stimuli to investigate their altered potential to induce an inflammatory response. Such approaches have been used to study inflammasome activation in monocytes of particle-exposed iron foundry workers (Westberg et al., 2019; Hedbrant et al., 2020) and TLR expression in dust-exposed pig farmers (Sahlander et al., 2010). However, the control populations are difficult to identify for evaluation of the results due to the heterogeneity in the healthy population with regards to inflammatory mediators and mechanisms and each experimental design needs thereby to provide its own controls.

#### In Vitro

To be able to scrutinize the molecular and cellular mechanisms involved in inflammatory responses to particle exposure, *in vitro* cell models are very useful. In addition to primary human cells, several cellular models of human origin are readily available, including the bronchial and alveolar epithelial cells: A549, BEAS-2B, NHBE, 16HBE14o-, HBE135-E6E7, H441, NCI-H292, Calu-3, NuLi-1, CuFi-1, and immunocompetent cells such as the monocytic cell lines THP-1 and U937 as well as primary cells isolated from healthy volunteers. All these models readily lend themselves to studies of cellular effects of particle exposure, including inflammatory potency in an airway relevant context. A study by Farcas et al. (2019) investigated the effect of emissions generated from a commercially available 3D printer inside a chamber, while operating for 1.5 h with ABS or PC filaments and collected in cell culture medium. Characterization of the culture medium revealed that repeat print runs with an identical filament result in the emission of various amounts of particles and VOCs. Mean particle sizes in cell culture medium were 201 ± 18 nm and 202 ± 8 nm for PC and ABS, respectively. At 24 h post-exposure, both PC and ABS emissions induced a dose dependent cytotoxicity, oxidative stress, apoptosis, necrosis, and production of proinflammatory cytokines and chemokines in human small airway epithelial cells (SAEC). Similarly, *in vitro* assays involving rat alveolar macrophages (NR8383, CRL 2192) and human tumorigenic lung epithelial cells (A549) showed toxic responses following the exposure to particles emitted from PLA and ABS. Interestingly, PLA-emitted particles elicited higher response levels than ABS-emitted particles at comparable mass doses (Zhang et al., 2019).

Furthermore, different culture strategies to better mimic the *in vivo* situation are available. To mimic the lung tissue, several strategies can be used, as reviewed by Heinen et al. (2021), including culture at the air-liquid interphase (ALI), fluidics systems to mimic shear stress, mechanical stretching systems to mimic breathing movement and organ-on-a-chip methods.

### Genotoxicity

Alterations of the genetic material can lead to severe health defects, as mutations in cells may elicit cancer and contribute to development of chronic diseases. Genotoxic events can occur at the DNA, chromosome, or whole genome level. Before genotoxicity testing, it is necessary to know the cytotoxicity of the tested particles and to establish the LC_50_ (lethal concentration at which 50% of cells die) in order to determine the appropriate range of exposure concentrations (Kohl et al., 2020). Due to the specific physicochemical features of particles, many standardized methods are inappropriate for the genotoxicity testing of particles. Thus, in the test strategy, the mammalian gene mutation test is recommended together with the micronucleus assay or γH2AX assay (Doak et al., 2012). In addition, different assays exist that assess intermediate endpoints, such as various types of DNA damage and novel markers measured with omics or epigenetics (Ventura et al., 2018). In recent years, there has been a strong effort to develop more complex, *in vivo*-like *in vitro* models based on 3D structures either of a single cell or co-cultures of two or more cell types. The application of these models in toxicology provides reliable data that are more relevant for evaluating genotoxicity in humans than standard single cell 2D culture models. Moreover, several existing genotoxicity testing methods, which are amenable to high content screening approaches have been identified (Collins et al., 2017).

The genotoxicity of particles and mechanisms leading to transient or permanent genetic alterations has been widely studied (Magdolenova et al., 2015; Doak and Dusinska, 2017; Nelson et al., 2017; Singh et al., 2019). Studies show that particle genotoxicity can result from two key mechanisms: primary (direct or indirect) or secondary genotoxicity. In primary genotoxicity, direct action of nanostructures on nucleic acids and induction of DNA damage could occur (Rezaei et al., 2021), whereas secondary genotoxicity usually implies a pathway of genetic damage resulting from the oxidative DNA attack by reactive oxygen/nitrogen species (ROS/RNS), generated during particle-elicited inflammation (Schins and Knaapen, 2007). For some particle types, one of these mechanisms might apply; however, for some particles both mechanisms can occur simultaneously. To define if primary genotoxicity is direct or indirect, it is crucial to understand the particle internalization mechanisms and the potential entrance into the nucleus. Particles of only a few nanometers in size can penetrate the nucleus *via* nuclear pores. Still, studies demonstrate the presence even of the larger particles in the nuclear compartment, showing that there could exist other pathways for nuclear internalization, e.g., intracellular processes resembling endocytosis (Kazimirova et al., 2020). During mitosis, the nuclear membrane is dissolved, allowing particles to enter the nucleus as it is reformed. When particles enter the nucleus, the interaction with DNA is considered a possible mechanism for direct genotoxicity. However, secondary genotoxicity is the main mechanism of particle genotoxicity, and it is mediated *via* ROS molecules produced by immune cells. Commonly, particles can trigger an oxidative burst when internalized by phagocytic cells. This event is an initial inflammatory/defense mechanism against invasion of non-self materials, including particles. However, in the case clearance of particles fails, it can lead to a chronic inflammatory response. Secondary genotoxicity cannot be studied with standard *in vitro* approaches and has so far only been investigated *in vivo* following chronic inflammation caused by activation of macrophages and/or neutrophils (Bartek et al., 2010). Importantly, shape, size, surface topography and chemical composition of the particle as well as the biotransformation and corona all play important roles in particle genotoxicity (Cowie et al., 2015; Xu et al., 2020). The same was confirmed in studies on genotoxicity of different metal particles (Vales et al., 2015; Rodriguez-Garraus et al., 2020). In the case of silver nanoparticles of same size genotoxicity and mutagenicity depend on their surface properties and charge (Huk et al., 2015). In addition, Gábelová et al. (2017) demonstrated that surface chemistry directly affects genotoxicity of iron oxide nanoparticles. Furthermore, (bio)transformation of particles, including chemical, physical, and biological processes could significantly affect the genotoxic potential of particles.

**FIGURE 6:**
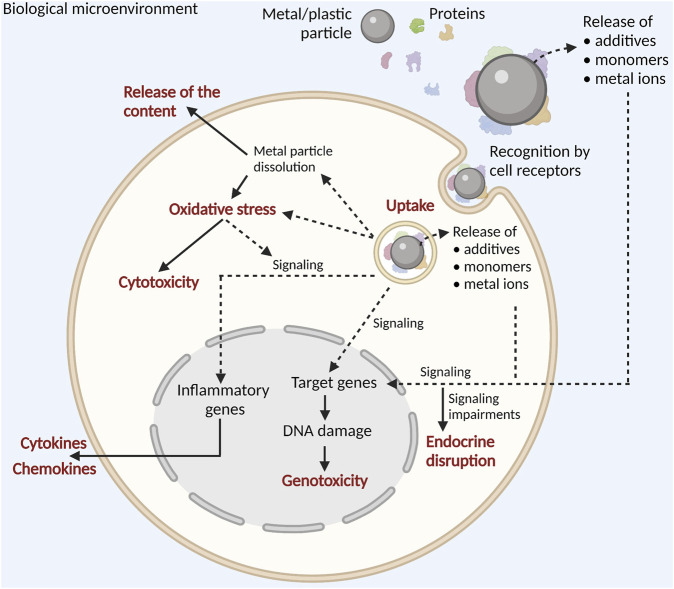
Particle-induced effects in the human cells. Both in the extracellular and intracellular milieu, particles potentially release additives, monomers, or metallic ions (depending on the particle chemical composition). After recognition and internalization, particles affect cell physiology in numerous ways by involving extensive signaling leading to changes in the expression of target genes, resulting in inflammation, endocrine disruption, genotoxicity, or cytotoxicity. Moreover, metal ions released during dissolution of metal particles may induce oxidative stress that may trigger inflammation and/or cytotoxicity.

Particle transformation during the AM processing is an important consideration in the AM particle safety assessment since AM powders are commonly reused, and as a result, their physicochemical features may be significantly altered, which in turn may vary their genotoxic potential. Additionally, formation and emission of mixed-size particles, like nanosized condensate particles or microsized spatter with very irregular shape, high degree of agglomeration, and increased surface oxidation may potentiate genotoxicity in the human cells. Finally, there is a huge gap in studying particle genotoxicity in relation to additives, monomers from polymer breakdown, metal ions, and biodegradation products that may be released from particles, both in the environment and in the target cells (Figure 6).

### Endocrine Disruption

Disruption of the endocrine function causes hormonal imbalance, influencing the development and occurrence of metabolic disorders. Most common approaches in assessing endocrine disruption involve *in vitro* screening assays designed to detect binding of disruptors to estrogen, androgen, glucocorticoid, aryl hydrocarbon (Ah), and peroxisome proliferator-activated receptors (PPARs). Currently available data support the notion that different types of particles can alter physiological activities of the endocrine tissues, as reviewed by Iavicoli et al. (2013). The reproductive systems of males and females are the endocrine organs that have received most attention. In the male reproductive system, particles can affect cell viability in gonadal tissues, testicular morphology, and the spermatogenesis. In the female reproductive system, particles cause toxic effects on ovarian structural cells and impaired oogenesis, and follicle maturation. In addition, particles elicit significant alterations in normal sex hormone levels both in females and males.

Additional important aspects of the possible roles of particles in the endocrine disruption are molecular mechanisms of action underlying the adverse effects. In several papers, oxidative stress was outlined as the main damaging mechanism (Zhu et al., 2009; Bai et al., 2010; Gao et al., 2012; Hou and Zhu, 2017). Also, silver nanoparticles have been found to inhibit cell proliferation by disrupting the proliferation signaling cascade of spermatogonial stem cells (Braydich-Stolle et al., 2010) with similar mechanism also observed for silicon carbide nanowires on ovarian cells (Jiang et al., 2010). Apart from those mechanisms, direct genotoxicity has been reported as an initiating event in endocrine disruption (Gromadzka-Ostrowska et al., 2012). For example, different studies confirm that particle-induced endocrine toxicity, very often, could occur due to particle mediated alteration of gene expression, leading to changes in activities of proteins and enzymes involved in sex hormone biosynthesis, metabolism, and release (Li et al., 2009; Gosso et al., 2011; Gao et al., 2012; Li et al., 2012). Additional hypothesis can be put forward, some endocrine disrupting particles could alter functions of the endocrine system due to their ability to bind to the hormone receptors (estrogen, androgen, thyroid receptors, etc.) and play a significant role in initiating adverse endocrine effects. For example, the estrogenic effects elicited in human cells may be dependent on particle interaction with various receptors, leading to activation/deactivation of the estrogenic signaling pathway. A study by Jain et al. (2012) reported that the release of ionic cadmium may contribute to the metalloestrogenic effects (estrogenic effect of a metal) of quantum dots (QDs) with a cadmium core. Study demonstrated that *in vitro* cadmium-containing QDs induce cellular proliferation, estrogen receptor α activation, and biphasic phosphorylation of protein kinase B (AKT) and extracellular signal-regulated kinases (ERK1/2), comparable with 17β-estradiol. These findings suggest that certain cadmium-containing nanocrystals are endocrine disruptors, with effects exceeding those induced by ionic cadmium or 17β-estradiol.

Apart from endocrine disruptive effects on sex hormones, particles may influence different metabolic pathways (Priyam et al., 2018). Gurevitch et al. (2012) demonstrated the potential role of titanium dioxide nanoparticles in the etiology of metabolic/endocrine disorders, such as obesity and insulin resistance. They showed that the exposure of Fao rat hepatoma cells to titanium dioxide nanoparticles altered insulin response and induced insulin resistance by interfering with insulin-signaling and by indirect inflammatory activation of macrophages.

In the AM environment, particles may cause endocrine disruption per se, but release of additives, monomers, metals, bound chemicals, and biodegradation products (especially from the polymeric/plastic particles) should not be neglected as an additional and so far, unexplored mechanism of inducing endocrine disruption, as shown in Figure 6. That issue should be properly addressed in order to complete a particle safety assessment framework and to move forward in the safer-by-design approach.

## Next-Generation Toxicology Tools for the Particle Safety Assessment

### High-Throughput Cell Profiling

Alongside the development of the classical toxicological endpoints and targeted assay for the evaluation of the toxicity, recent advances in the development of a non-targeted assay called Cell Painting (Bray et al., 2016) are making this technique a promising tool for toxicological studies.

Cell Painting assay employs a set of fluorescent dyes to stain the cells’ different compartments. Cells are treated in multi-well plates with the chemicals of interest (one chemical per well), at one or several concentrations of each tested chemical, for 24–48 h. After fixation and staining, fluorescent images of the cells are captured at the relevant for each dye, wavelengths using a high-content screening system. CellProfiler (https://cellprofiler.org/), a cell image analysis freeware developed by the Carpenter Lab at Broad Institute, is used to measure ≈3200 morphological features, like size, shape, texture, intensity, etc. from the individual cells, thus producing complex feature profiles for each treatment (Carpenter et al., 2006; McQuin et al., 2018). Extracted morphological profiles can be used for the characterization of the chemicals, and for elucidation of their mechanisms of action.

Screening of 462 environmental chemicals from the ToxCast library in a concentration-response mode using Cell Painting assay with U-2 OS cells showed that approximately two-thirds of the tested chemicals were administered at a concentration dose that is comparable to the corresponding *in vivo* potency. The rest of the chemicals over-predicted the *in vivo* dose, which could be partially explained by the lacking expression of the molecular targets for those chemicals in U-2 OS cells (Nyffeler et al., 2020). Considering the last limitation of the U-2 OS cells, other cell lines may be used and are being tested with this assay (Bray et al., 2016; Willis et al., 2020). The combination of phenotypical profiles with other descriptors, like chemical descriptors, gene expression, knock-out data, etc., and with machine learning algorithms for image characterization and clustering increases predictivity of the chemical toxicity by the Cell Painting assay (Chavan et al., 2020; Way et al., 2021).

Interaction of cells with particles has an impact on the cell morphology (Lipski et al., 2008), thus the application of high-content screening by employing approaches, like Cell Painting, to study the toxicity and mechanisms of particle action is of great interest. Complementing high-content screening with additional methods (e.g., omics technologies and machine learning) offers an excellent tool for a detailed deconvolution of particle mechanisms of action in cells. Recently, we have successfully applied Cell Painting integrated with lipidomics, metabolomics and unsupervised learning for the detection of cell response signatures to (nano)particles released in metal AM (unpublished data).

### Multiomics Testing Strategies

The omics technologies include transcriptomics, proteomics, metabolomics, epigenomics, and genomics. By using these techniques, it is possible to identify, not only toxic but also the adaptive responses to toxicants at low exposure levels putting cells or organisms under stress or inflammation, which is often the case with low-dose and chronic particle exposures. Early identification of altered cellular conditions at low doses is crucial for elucidating mechanisms of action as the toxicity will occur when the compensation and repair systems are exhausted. Particles usually interact with multiple structures and biomolecules in the cells and perturbate multiple signaling pathways impacting different cellular processes, including proliferation, cell death, oxidative stress, inflammation, and membrane integrity, and it is usually difficult to deduce the pathway of toxicity from the regulation pattern (Fröhlich, 2017; Hartung et al., 2017). Hence, systems toxicology integrates a system-wide response, such as transcriptomics, metabolomics, and proteomics data to capture a variety of omics data and metadata to comprehensively address particle toxicity.

The transcriptome consists of the entire set of transcripts or mRNAs present in a cell or an organism. Gene expression profiling outlines the expression level of mRNAs at a given time point by DNA microarrays, next generation RNA sequencing, subtraction hybridization, differential display, or serial analysis of gene expression. Even so, a known limitation of transcriptomics lies in the fact that changes in gene expression do not correlate directly with the phenotypic changes (Nikota et al., 2015; Fröhlich, 2017). Proteomics defines the analysis of functionally and structurally related proteins, thereby providing more direct information on cellular responses than transcriptomics, and can capture non-transcriptionally regulated responses, including fast modification or changes in cellular localization of proteins. Proteomics also plays an important role in identification of regulated pathways and identification of proteins adsorbed to the particle’s surface (protein corona) (Monopoli et al., 2011; Vogt et al., 2015). In comparison to transcriptomics and proteomics, which provide information of potential particle hazards, metabolomics directly identifies phenotypic changes that occurred under particle exposure by analyzing variations in carbohydrate, lipid, and amino acid patterns. Metabolomics is not organism-specific and does not have a fixed code (Ramirez et al., 2013), offering the great opportunity to profile the entire metabolome either as a footprint (extracellular metabolites) or as a fingerprint (intracellular metabolites).

Numerous *in vitro* and *in vivo* studies have assessed particle effects by omics techniques. Table 2 summarizes some of the *in vitro* studies by using an omics approach, with a focus on lung and immune cell models. These studies emphasize the importance of pre-condition analyses prior to the omics, including a simple and straightforward cytotoxicity screening to determine the particle concentration range. Such approach is crucial because highly cytotoxic particle concentrations should be avoided as apoptotic/necrotic cells provide only limited information on particle regulatory mechanisms. Fröhlich (2017) summarizes limitations that hinder the broad use of omics technologies in particle toxicology, including lack of standardized methods for particle exposure (sample pre-treatment, cell type, complex medium composition) and relevant concentration range. Such limitations should be taken into consideration when designing and performing particle toxicity studies.

**TABLE 2:** Brief overview of the *in vitro* studies characterizing the effects of particles on the human cell transcriptome, proteome, and metabolome.

Omics technique	Particle type	Experimental model(s)	Dose and timepoint	Regulated pathway(s)	References
Transcriptomics	Iron oxide (Fe_3_O_4_)	RAW264.7, Hepa1–6	30–100 μg/ml; 4–48 h	Immune effects, cell death, homeostatic processes	Liu et al. (2014)
	Silicon dioxide (SiO_2_)	A549	50–600 μg/ml; 2 h	Inflammation, apoptosis, matrix metalloproteinases	Fede et al. (2014)
	Iron oxide (Fe_3_O_4_)	KG1a, HL60	50 μg/ml; 72 h	Lipid metabolism, antioxidation, Hypoxia-inducible factor-1 (HIF-1) signaling pathways	Luo et al. (2020)
Proteomics	Titanium dioxide (TiO_2_)	BEAS-2B	10 μg/ml; 24 h	Stress response, metabolism, adhesion, cytoskeleton dynamics, cell growth, cell death, cell signaling	Ge et al. (2011)
	Silicon dioxide (SiO_2_)	A549	100 μg/ml; 24 h	Apoptosis, cytoskeleton, oxidative stress response, protein synthesis	Okoturo-Evans et al. (2013)
	Silicon dioxide (SiO_2_)	A549	0.1–6 μg/cm^2^, 24 and 72 h	Rho signaling cascade, cytoskeleton remodeling, endocytosis, inflammation, coagulation system pathway, oxidative stress	Pisani et al. (2015)
Metabolomics	Aluminum oxide (Al_2_O_3_)	HBE	50–500 μg/ml; 24 h	Apoptosis, oxidative stress, mitochondrial function	Li et al. (2015)
	Copper oxide (CuO)	A549	5–40 μg/ml; 4–24 h	Oxidative stress, hypertonic stress, apoptosis	Boyles et al. (2015)
	Carbon black nanoparticles (CBNPs)	A549	70 μg/ml, 48 h	Energy, amino acid, and lipid metabolism	Hou et al. (2020)

### Machine Learning

Increasing number of simple and composite particulate materials is produced in various conformations (Winkler and Le, 2017; Lamon et al., 2019). Particle safety assessment is extremely time-, labor-, animal-, and cost-consuming (Nel et al., 2013). Large and complex datasets generated in high-content screening assays and omics studies are ideal for modeling and analysis by modern machine learning methods. For example, unsupervised machine learning may reduce dimensionality of the Cell Painting and omics datasets. This will provide visual representation of the secondary data analysis that will be helpful in predicting similar mechanisms of action and classification of the particles based on their hazardous potential. In addition, machine-identified cell states will reveal how particle-specific effects correspond to the known disease states and known mechanisms of the toxin action. In addition, data driven modeling techniques based on supervised machine learning have concurrently risen in importance, sophistication, and utility, driven largely by the availability of these massive datasets. Larger training datasets usually result in models with high prediction accuracies and large domains of applicability (Winkler, 2020).

The physicochemical characteristics of particles must be encoded as mathematical entities called descriptors that are used by machine learning to generate predictive models (Young et al., 2012; Mikulskis et al., 2019). Particles have certain issues that make finding useful descriptors more difficult than for single molecules/chemicals. These depend on the size and shape distribution of particles, their tendency to aggregate/agglomerate, and affinity to interact with biomolecules creating corona that modulates the biological characteristics of particles (Ke et al., 2017). Commonly, particle descriptors are obtained from the whole particle and include particle diameter, surface, aspect ratio, number of atoms, number of surface atoms, potential energy of surface atoms, descriptors for surface coating, zeta potential, solubility, etc. (Epa et al., 2012; Winkler, 2020). Traditional machine learning methods include linear and nonlinear regression, artificial neural networks, various types of decision trees (Svetnik et al., 2003), Bayesian networks (Aguilera et al., 2011), support and relevance vector machines (Burden and Winkler, 2015), and genetic algorithms (Winkler and Le, 2017). For example, Liu et al. (2013) reported classification machine learning models of the effect of 44 iron oxide core nanoparticles on aortic endothelial, vascular smooth muscle, hepatocyte, and monocyte/macrophage cells, using four different biological assays. Their two-class models had relatively high accuracies >78%. Recently, a study by Huang et al. (2020) reported use of a Quantitative Structure-Activity Relationship (QSAR) models to predict IL-1β release in THP-1 cells following the exposure to metal oxide nanoparticles. QSAR models based on IL-1β were able to predict the inflammatory potential of nanoparticles allowing for computational assessment of particles’ inflammatory potential depending on their physicochemical characteristics.

Importantly, machine learning approaches are critically dependent on large datasets for model training and validation, generation of relevant descriptors that represent physicochemical properties of particles, robust training of models, and use of the models to predict characteristics of novel materials (Winkler, 2020). By this in mind, machine learning models hold an excellent potential for the safer-by-design approach when designing novel particulate materials, including feedstocks applied in AM.

### Adverse Outcome Pathway Networks as a Promising Tool for the Particle Safety Assessment

**FIGURE 7:**
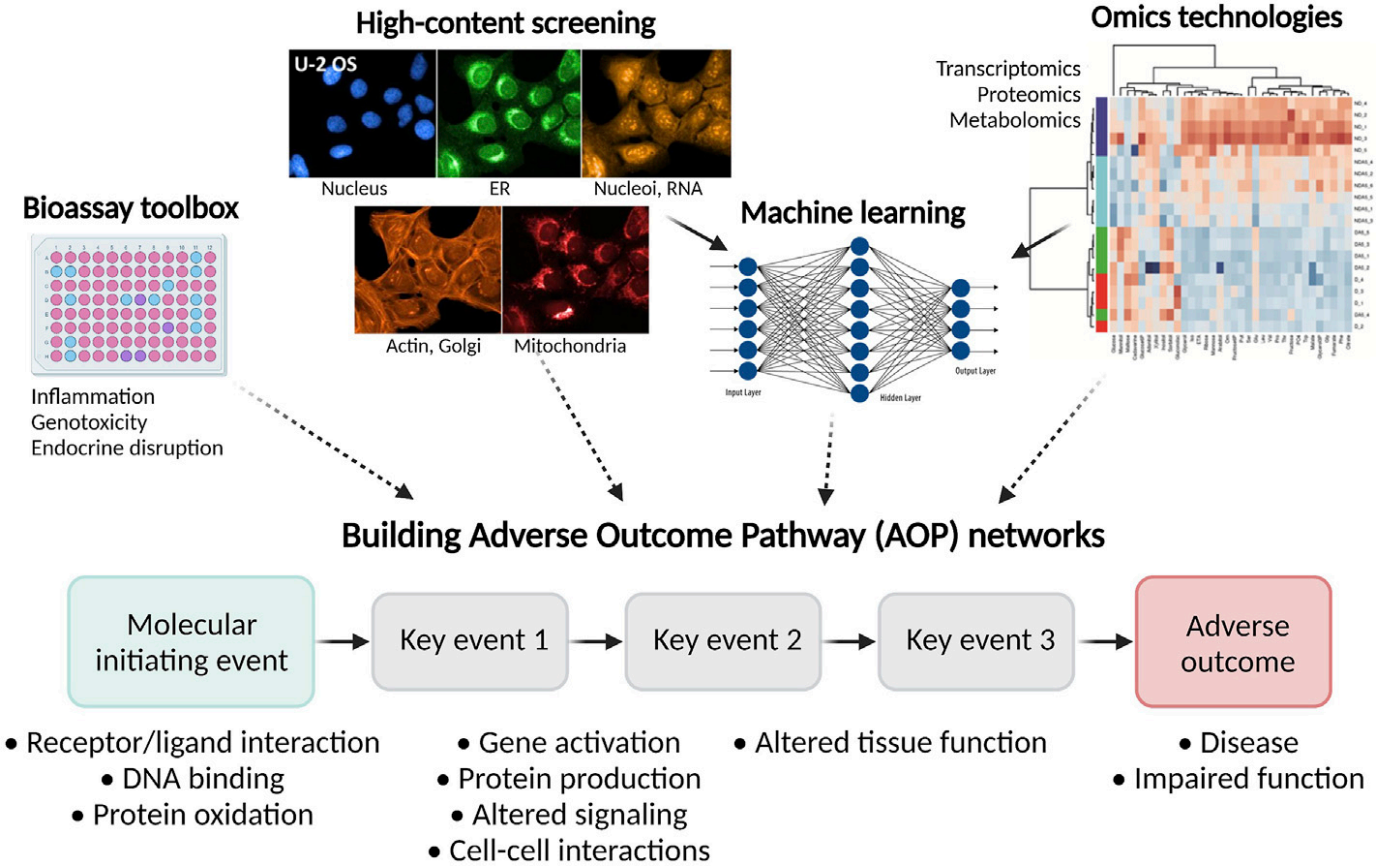
Next-generation toxicology testing for the particle safety assessment. Integration of the bioassay toolbox, high-content screening, omics technologies, and machine learning models offers excellent input in the development of AOP networks and description of MIEs, KEs, and AOs.

Large datasets obtained in *in vitro* assays, high-content screening, and omics studies are excellent inputs to build adverse outcome pathway (AOP) networks (Figure 7). When first described in the context of ecotoxicological risk assessment, an AOP was defined as “a conceptual construct that portrays existing knowledge concerning the linkage between a direct molecular initiating event (MIE) and an adverse outcome (AO),” by capturing the sequential chain of causally linked Key Events (KEs) at different levels of biological organization—from molecules to organism level (Vinken, 2018; Halappanavar et al., 2020; Nymark et al., 2021). Detailed guidelines are established by the OECD (2016), and a large database of AOPs describing various adverse outcomes of relevance to human health is available (https://aopkb.oecd.org/). Recently, AOP development in the field of particle toxicology has particularly focused on case study-based and data mining approaches, with the aim to develop new AOPs or refine existing AOPs based on one or several model stressors. The data mining approach is employed when there is sufficient high-content and high-throughput information, such as omics data, available to identify KEs and to support the development of AOPs (Gerloff et al., 2017; Nymark et al., 2018; Halappanavar et al., 2020).

In general, AOPs developed for chemicals should be applicable to particles (Halappanavar et al., 2020). Different biological pathways and adverse outcomes (AO) induced by nanoparticles are shown to share similarities with those triggered by chemicals, albeit with a lack of detailed understanding of the MIE (Gerloff et al., 2017). Mechanistically, when deposited in the lungs, particles induce oxidative stress, inflammation, genotoxicity, and cytotoxicity (Oberdörster et al., 2007). Metal oxide nanoparticles are demonstrated to induce toxic effects similar to specific occupational hazards (e.g., silica) (Napierska et al., 2010). Therefore, in principle, toxicity pathways and key biological events describing chemical-induced AOs should be applicable in the case of particles.

An example of an AOP network is the development of lung cancer due to the occupational exposure to fibers and particles, such as asbestos (Nymark et al., 2008). The underlying mechanism is initiated by the interaction of particles with lung macrophages; leading to frustrated phagocytosis. This event is described as the MIE in this AOP network. Furthermore, frustrated phagocytosis, and consequential biopersistence of particles, causes lung inflammation, characterized by increased release of proinflammatory cytokines (this is key event 1 (KE1)) and by increased invasion of leukocytes into lungs (this is KE2). Intensified cytokine release and functional alterations of the immune cells lead to an increased production of ROS (this is KE3) (Mittal et al., 2014). The ROS is acting as a secondary messenger and mediator of inflammation leading to DNA damage and gene mutation (this is KE4) in lung epithelial cells (Hiraku et al., 2015). Proliferation (this is KE5) is a highly surveilled process that maintains tissue homeostasis. However, when proliferation checkpoints are impaired, an increase of proliferation is detected, which is one of the hallmarks of cancer (Hanahan and Weinberg, 2011). The uncontrolled proliferation of lung cells usually evokes increase of mutations in oncogenes or tumor suppressors, and inevitably induce the development of cancer (this is the AO).

Importantly, AOP networks offer a great next-generation toxicology tool for particle safety assessment that will enable deeper understanding of mechanisms involved in particle toxicity. More precisely, AOP networks can be used to inform the design and development of targeted *in vitro* assays, and for generating quality data necessary for human health risk assessment of particles (Halappanavar et al., 2020).

## Conclusion and Outlooks

AM has entered in a dynamic development phase in the industry. Feedstock powders and particles formed and emitted during AM processes may pose potential occupational risk for the workers and potentially end users. To mitigate risks, it is important to design comprehensive safety assessment framework that will map particle exposures, characterize captured particles, understand mechanisms by which particles act to induce adverse effects, and to translate findings into an *in vivo* setting and identify long-term and low-dose exposure health effects. However, particle safety assessment is facing challenges in collection of the airborne particle emissions in the AM facilities. Due to small size and technical limitations of the collection instruments, it is very hard to collect enough nanosized particles for toxicological testing. While optimizing these methods, safety assessments should focus on feedstock powders, impact of powder reuse, and particles captured into air-ventilation filters that can be recovered in sufficient quantities for biological testing. To address biological effects of AM particles, a completely new approach of next-generation toxicology testing is emerging. It includes a systemic approach with advanced *in vitro* and *in silico* models, high-content screening, omics technologies, big data approaches, and development of AOPs as mechanistic tools in particle safety assessment. The authors of this article, consider this review an opportunity to: 1) highlight particle exposure-related risks in AM, 2) to define well-established toxicological endpoints relevant for AM particle assessment, and 3) to propose next-generation toxicology testing approaches. Together, the proposed approach will enable a deeper understanding of existing and emerging particle and chemical safety challenges and provide a strategy for the development of cutting-edge methodologies for hazard identification and risk assessment in the AM industry.

## Glossary

AM: additive manufacturing
3D printing: three-dimensional printing
L-PBF: laser powder bed fusion
SRA: Strategic Research Agenda
VOCs: volatile organic chemicals
SLM: selective laser sintering
EBM: electron beam melting
CAD: computer-aided design
OEL: occupational exposure limit
MJ: material jetting
ME: material extrusion
FDM: fused deposition modeling
PLA: polylactic acid
PS: polystyrene
ABS: acrylonitrile butadiene styrene
PRR: pattern recognition receptors
SR: scavenger receptor
NLR: NOD-like receptor
TLR: Toll-like receptor
CVD: cardiovascular disease
IL-1β: Interleukin-1β
IL-18: Interleukin-18
NET: neutrophil extracellular trap
LMR: lymphocyte-to-monocyte ratio
SAEC: small airway epithelial cells
CRP: C-reactive protein
ROS: reactive oxygen species
RNS: reactive nitrogen species
FeNO: fractional exhaled nitric oxide
NO: nitric oxide
CC16: Club cell secretory protein 16
ALI: air-liquid interphase
ERK1/2: extracellular signal-regulated kinases 1/2
Ah-receptor: aryl hydrocarbon receptor
PPAR: peroxisome proliferator-activated receptors
QD: quantum dot
QSAR: Quantitative Structure-Activity Relationship
AOP: adverse outcome pathway
MIE: molecular initiating event
KE: key event
AO: adverse outcome.
